# State of the “Art” in Precision Health Symptom Science Research

**DOI:** 10.1016/j.soncn.2025.151906

**Published:** 2025-05-30

**Authors:** Christine Miaskowski, Sara Colomer-Lahiguera, Yvette P. Conley, Susan Dorsey, Marilyn J. Hammer, Carolyn Harris, Marques Shek Nam Ng, Michele Pelter, Nancy Redeker, Susan Wesmiller

**Affiliations:** a School of Nursing, University of California, San Francisco; b School of Medicine, University of California, San Francisco; c Faculty of Biology and Medicine, University of Lausanne, Switzerland; d School of Nursing, University of Pittsburgh, Pennsylvania; e School of Nursing, University of Maryland, Baltimore; f Phyllis F. Cantor Center, Dana Farber Cancer Institute, Boston, Massachusetts; g Nethersole School of Nursing, The Chinese University of Hong Kong, Shatin, New Territories, Hong Kong; h School of Nursing, University of Connecticut, Storrs

**Keywords:** Artificial intelligence, Exploratory factor analysis, Latent variable modeling, Machine learning, Network analysis, Symptoms

## Abstract

**Objectives:**

The purposes of this paper are to provide a historical perspective on precision health symptom science research; discuss current “conundrums” in precision health symptom science research, including gaps in knowledge and opportunities for growth; and highlight potential challenges that could impede the advancement of precision health symptom science research. The paper concludes with critical directions and recommendations for future research.

**Methods:**

A narrative review of the literature on symptoms and symptom burden, across common medical conditions (ie, cancer, heart failure, chronic obstructive pulmonary disease, chronic kidney disease, human immunodeficiency virus, type 2 diabetes mellitus, was conducted to provide information on salient topics associated with precision health symptom science research.

**Results:**

This paper provides an overview of the field of precision health symptom science research. Some of the conundrums discussed include measurement of single and multiple symptoms; interindividual variability in persons’ symptom experiences; the concept of “time” in the evaluation of symptoms; advantages and disadvantages of various analytic approaches; and the collection of “meaningful” data. Practical research examples and suggestions are included to guide the development and conduct of future studies.

**Conclusions:**

The paper summarizes potential challenges and opportunities in precision health symptom science research. The final section of the paper provides a summary of critical directions and recommendations for future research.

**Implications for Nursing Practice:**

Precision health symptom science is a relatively young field of scientific inquiry. However, the future of precision health symptom science research is both exciting and challenging. Knowledge gained in this field will result in the identification of risk factors for a higher symptom burden and the development and testing of personalized interventions to prevent and/or treat symptoms across one or more acute or chronic medical conditions.

A symptom is defined as “something that a person feels or experiences that may indicate that they have a disease or condition. Symptoms can only be reported by the person experiencing them. They cannot be observed by a health care provider or other person and do not show up on medical tests.”^1^ The importance of research into all aspects of symptoms (eg, epidemiology, measurement, underlying mechanisms, impact, management) cannot be overemphasized. This statement is justified because, across all chronic medical conditions, individuals with a higher symptom burden report significant decrements in: work-related activities^2^; engagement in meaningful social relationships^3^; psychological health and well-being^4^; functional status^5^; and the performance of daily activities.^6^ In addition, this burden is associated with decreased adherence with treatment regimens^7,8^; significant financial toxicity^9^; and in some cases, increases in mortality.^10^ While the foundation for precision health symptom science research comes primarily from studies of patients with cancer,^11^ a growing body of evidence supports the negative consequences of multiple co-occurring symptoms in patients with a variety of chronic conditions (eg, heart failure [HF],^12^ chronic obstructive pulmonary disease [COPD],^13^ human immunodeficiency virus,^14^ chronic kidney disease,^15^ type 2 diabetes mellitus^16^).

The purposes of this paper in this Special Issue of *Seminars in Oncology Nursing* are to provide a historical perspective on precision health symptom science research; discuss current “conundrums” in precision health symptom science research including gaps in knowledge and opportunities for growth; and highlight potential challenges that could impede the advancement of precision health symptom science research. The paper concludes with critical directions and recommendations for future research.

## A Historical Perspective on Precision Health Symptom Science Research

One may consider that precision health symptom science research began with studies of pain^17,18^ and fatigue^19,20^ in patients with cancer in the 1980s and 1990s, respectively. However, it is worth noting that Florence Nightingale in her Notes on Nursing, published in 1859, provided a clear statement on the role of the nurse in the assessment and management of symptoms. In this ground-breaking treatise, Nightingale states “*the most important practical lesson that can be given to nurses is to teach them what to observe*—*how to observe*—*what symptoms indicate improvement*—*what the reverse*—*which are of importance*—*which are of none*—*which are the evidence of neglect*—*and of what kind of neglect*” (p. 59).^21^

While the word symptom appears only once in Notes on Nursing,^21^ this quote emphasizes the need for comprehensive assessment of symptoms and longitudinal evaluations of changes in an individual’s symptom burden and responses to treatments. In addition, Nightingale suggests that an evaluation of the contribution of social determinants of health (SDOH) to symptom burden is extremely important and that interindividual differences exist in persons’ symptom experiences. A true visionary and well ahead of her time, Nightingale recognized some of the key elements of rigorous precision health symptom science research.

Fast forward to 2019, when a group of nurse scientists published a paper that clarified the terms “symptom science” and “precision symptom science.”^22^ In this paper, the authors noted that the goal of symptom science research “*is to be able to precisely identify individuals at risk for symptoms and develop targeted strategies to prevent or mitigate the severity of symptoms*” (pp. 86–87). In contrast, precision symptom science holds “*a perspective that includes not only the* ‘*omic*’ *but also the social, societal, and environmental determinants of health. The ultimate purpose of this initiative is to improve the health of patients through a more comprehensive understanding of the factors that interact to contribute to symptoms [N.B. in the publication the word pain is used], with this evidence being essential to develop precision-based interventions for symptom(s)*” (p. 87). These definitions lay the foundations for precision health symptom science research that include: an evaluation of interindividual differences in persons’ symptom experiences; identification of a variety of modifiable and nonmodifiable risk factors associated with a higher symptom burden; the use of biomarkers to determine the underlying mechanisms for symptoms; determination of the impact of symptoms on patient outcomes; and the development and testing of precision-based interventions to prevent symptoms and/or decrease symptom burden. From a historical perspective, the next few decades represent a critical time in precision health symptom science research. Given recent advances in assessing symptom phenotype(s), the integration of symptom phenotypes with a variety of biomarkers; the availability of large amounts of data arising from digital health applications, and advances in analytic methods, precision health symptom scientists are on the precipice of making significant breakthroughs in the assessment and management of common symptoms that occur across a variety of common medical conditions.

## Current Conundrums in Precision Health Symptom Science Research

A conundrum is defined as a “confusing or difficult problem or question” or “an intricate and difficult problem.”^23^ In common usage, the word is often linked with solving a puzzle. In this paper, the following conundrums will be discussed: measurement of symptoms; evaluation of interindividual variability in persons’ symptom experiences; the concept of “time” in the evaluation of symptoms; useful approaches to analyze symptom data; and the collection of “meaningful” data. While not an exhaustive list, these conundrums warrant careful consideration to be able to advance this important area of scientific inquiry.

### Measurement of Symptoms

A quote from Lord Kelvin, in a lecture that he gave to the Institute of Civil Engineers in 1883, provides meaningful instruction on measurement. He stated that “*To measure is to know. I often say that when you can measure what you are speaking about, and express it in numbers, you know something about it; but when you cannot measure it, when you cannot express it in numbers, your knowledge is of a meagre and unsatisfactory kind; it may be the beginning of knowledge, but you can scarcely in your thoughts advance the states of the science, whatever the matter may be*.”^24^

#### Considerations in choosing a symptom(s)—

The first step in the development of a plan for precision health symptom science research is to decide on the symptom(s) that will be the focus/foci of the study. One needs to decide on whether to study a single symptom or multiple concurrent symptoms. In terms of a single-symptom study, some of the guiding principles for choosing a single symptom include: the patient population under investigation; the prevalence of the symptom; the state of the science and current gaps in knowledge about the symptom; the impact of the symptom on patient outcomes; and the availability of funding and associated resources.

#### Evaluation of single symptoms—

However, the study of single symptoms is not without its challenges. One conundrum is whether to study a “common” symptom versus a “less” common or “emerging” symptom. As shown in Table 1, across a variety of common medical conditions, the most common symptoms reported by individuals are: fatigue, pain, sleep disturbance, cognitive changes, and depression.^11,13–16,25–27^ If one chooses a common symptom, usually defined by its high prevalence rate and its association with significant negative outcomes, the gaps in knowledge about this symptom must be stated explicitly. For example, an investigation of the correlations between subjective (ie, self-report) and objective measures of a symptom (eg, cognitive impairment, sleep disturbance) and similarities and differences in risk factors and/or underlying mechanisms may be warranted to develop targeted interventions.

If an “emerging” symptom is the focus of the investigation, the scientific justification inherent in this choice is the lack of knowledge of the symptom’s epidemiology, associated risk factors, underlying mechanisms, current management approaches, and their efficacy, and/or deleterious outcomes. In patients with cancer, some “emerging” symptoms include: hearing loss, tinnitus, skin changes associated with targeted therapies, dyspnea, palpitations, and diarrhea. The identification of “emerging” symptoms may come from knowledge of the adverse effects associated with novel therapeutics being used to manage a variety of acute and or chronic medical conditions, and/or individuals’ reports of their symptom experience.

For both “common” and “emerging” symptoms, one of the most challenging aspects of any study is choosing a measure (ie, instrument, questionnaire, scale) to assess the symptom. For many “common” symptoms, an instrument “soup” exists. For example, as noted in systematic reviews, 39 valid and reliable self-report measures are available to evaluate fatigue^28^ and 22 measures are available to evaluate sleep disturbance.^29^ Things to consider in choosing an instrument beyond its validity and reliability include: administration time; availability in multiple languages; participant challenges with interpretation of the items on the measure and/or associated rating scales; recall period; sensitivity to detect changes in the symptom over time; and the availability of clinically meaningful cutoff scores.

Another consideration in the evaluation of single symptoms is the dimension(s) of the symptom experience that will be evaluated. Some of the most common dimensions of the symptom experience include: occurrence, severity, frequency, distress, and interference. The rationale for assessing multiple dimensions of the symptom experience comes from studies that demonstrated that the most common symptoms are not always the most severe and/or distressing. In addition, evidence suggests that the most severe symptoms are not always the most distressing and vice versa.^11,30^

#### Evaluation of multiple symptoms—

In making the choice to study multiple co-occurring symptoms, one needs to ask the question—“Is more better?”. Based on the clinical reality—the answer to this question is an unequivocal YES! As shown in Table 2, across most of the common medical conditions, in the “real world,” individuals present with more than one symptom. Similar to a single-symptom study, the choice of a measure to evaluate multiple symptoms warrants careful consideration. Potential measures should be compared in terms of the number of symptoms included in the measure; the dimension(s) of the symptom experience that are assessed; the conceptual clarity of the measure; the number or translations; the time for completion; its specificity for a particular medical condition, and its ability to detect changes in symptom burden over time. For example, in studies of patients with cancer, the Memorial Symptom Assessment Scale contains 32 symptoms; evaluates symptom occurrence, severity, frequency and distress, and is available in 23 languages.^31^ In contrast, the MD Anderson Symptom Inventory contains 13 symptoms, evaluates symptom severity, and is available in 48 languages.^32^ Of note, the number of symptoms on a given inventory will impact the findings of a study (eg, number of symptom clusters that can be identified).

### Interindividual Variability in Persons’ Symptom Experiences

**FIG 1.**
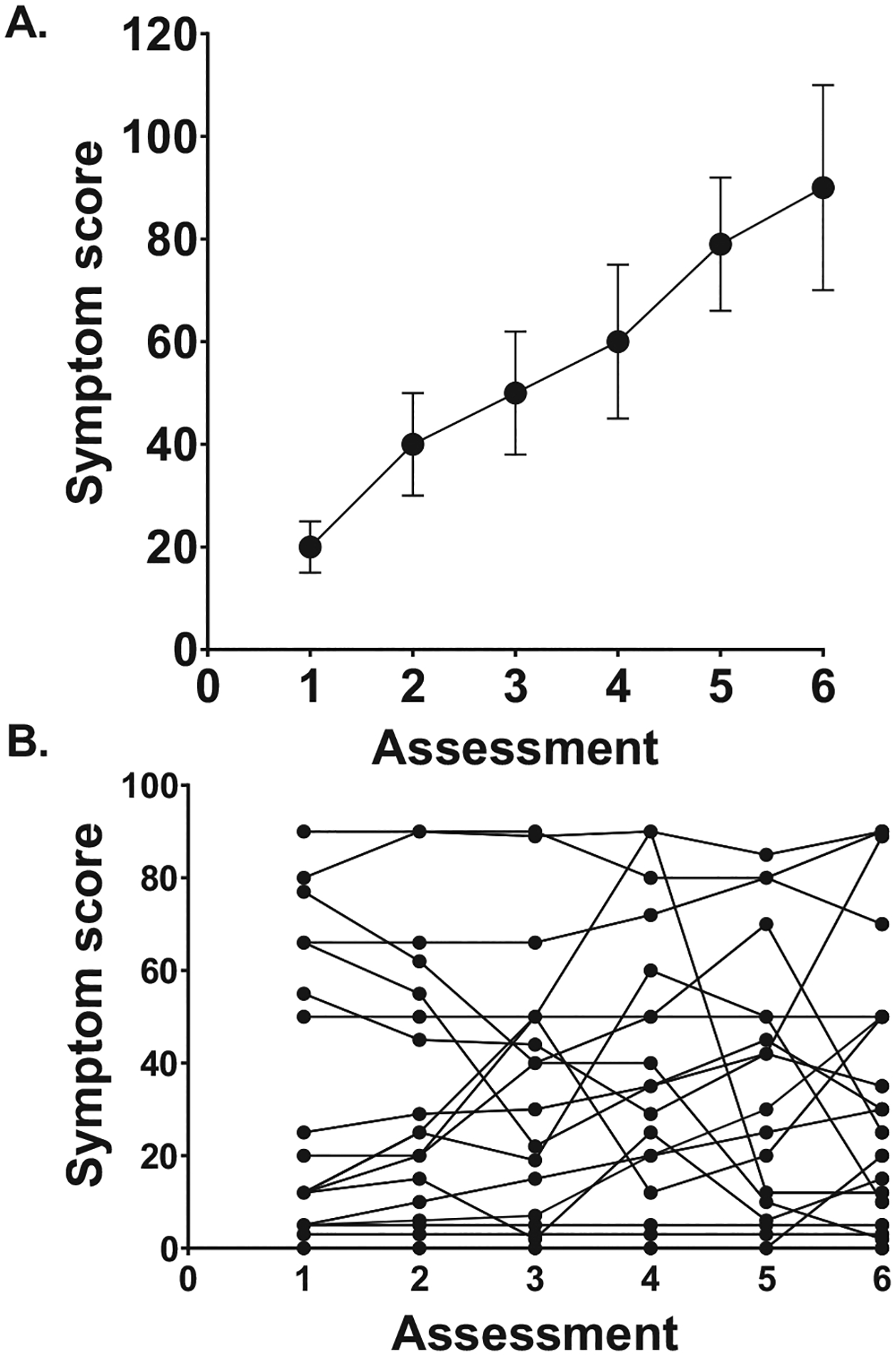
(A) An illustration of the “older” or more traditional approach that is used to evaluate changes in a symptom over time. Changes over time in a symptom score are plotted as means and standard deviations at each assessment. (B) An illustration of the amount of interindividual variability in a symptom score. Individual symptom scores for 38 participants over six assessments are plotted on the figure.

Another conundrum in precision health symptom science research is how to evaluate for interindividual variability in persons’ symptom experiences. As shown in Fig. 1A, the “older” or more traditional approach to the evaluation of changes in symptoms over time was to use analysis of variance and to illustrate these changes in occurrence rates using percentages and/or changes in symptom severity or distress scores using means and standard deviations. However, in reality, for any given symptom within any study sample, a large amount of interindividual variability exists in its occurrence rates and/or in its severity or distress scores (Fig. 1B). Specifically, some individuals with the same medical condition may report very few symptoms, as well as low levels of severity and distress. In contrast, other individuals may report the maximum number of symptoms with very high levels of symptom severity and/or distress. This fact emphasizes the need for and importance of precision health symptom science research.

In the era of precision health symptom science research, some of the salient questions are: (1) how best to identify individuals with a lower versus a higher symptom burden; (2) how best to identify modifiable and nonmodifiable risk factors associated with a higher symptom burden (eg, demographic and clinical characteristics; SDOH, stress, loneliness; biomarkers); (3) how best to determine the deleterious outcomes of a higher symptom burden; (4) how best to determine the mechanisms that underlie a higher symptom burden; and (5) how best to design and test personalized interventions for individuals with the highest symptom burden. The subsequent section entitled “useful analytic approaches to evaluated interindividual variability and associated risk factors” provides some suggestions on how to evaluate for interindividual variability in persons’ symptom experiences using single and multiple symptoms.

### Concept of “Time” in the Evaluation of Symptoms

When doing precision health symptom science research, investigators often think about time in terms of how long it will take to complete study questionnaires; how long will it take to complete the entire study; and how long will it take to analyze the data and disseminate the findings. However, additional considerations are warranted as part of the design of a precision health symptom science study.

First and foremost, an investigator needs to determine the scope of the symptom assessment. How comprehensive should the assessment be? What trade-offs need to be made in terms of depth versus breadth? In other words, what is the best approach to characterize the symptom phenotype? For example, in conducting a study on chemotherapy-induced peripheral neuropathy (CIPN), some relatively brief and valid and reliable measures include: the European Organization for Research and Treatment of Cancer Quality of Life Questionnaire-CIPN 20 (20 items),^33^ the Functional Assessment of Cancer Therapy/Gynecological Cancer Group Neurotoxicity Questionnaire (11 items),^34^ and the Total Neuropathy Score (assesses 3 sensory and 2 motor neuropathy symptoms, as well as vibration, strength, and deep tendon reflexes).^35^ Alternatively, an investigator can choose to do a more in-depth evaluation of CIPN using the Brief Pain Inventory,^36^ the Pain Qualities Assessment Scale,^37^ a body map of the hands and feet, an assessment of aggravating and relieving factors, and an assessment of the frequency of use and efficacy of any number of pharmacologic and nonpharmacologic interventions. The scope of the symptom assessment is linked to the specific research question(s); the level of current knowledge of a particular symptom phenotype; as well as the amount of time to complete the measures and the inherent burden on the participant.

Second, the selection of the appropriate recall period is another “time” conundrum. The choice of recall period (eg, now, past 24 hours, past week) depends on the study’s specific aims, as well as the person’s medical condition and associated treatments. While short recall periods can mitigate memory bias, longer ones can result in recall bias or the omission of symptoms. The accuracy of a person’s recall depends on the complexity and saliency of the symptom(s), as well as the person’s previous experiences and mood.^38^ This conundrum warrants careful consideration to ensure reliable results.

Another time conundrum is the timing of the administration of the symptom measure(s). In some studies, the administration of the symptom measure(s) can be linked to critical times in the trajectory of the individual’s medical condition (eg, time of diagnosis, initiation of a specific treatment, transitions in care). However, during stable periods in an acute or chronic medical condition (eg, cancer survivorship, maintenance dialysis), the appropriate timing for the administration of study measures may be less clear and may need to be guided by participants’ level of tolerance for completing the repeated measures (eg, once a month, three times a year, quarterly).

A major gap in knowledge exists about the optimal timing of various symptom measures within the context of the most common acute and chronic medical conditions to be able to capture changes in symptom burden. Increased knowledge is needed on when a symptom would be expected to change during the course of clinical care. Equally important, in intervention studies, additional research is needed to optimize the timing of questionnaire administration to be able to determine when treatment efficacy occurs and if it is sustained.

### Useful Analytic Approaches to Evaluate Interindividual Variability and Associated Risk Factors

Analytic approaches in precision health symptom science range from the simple to the complex and are dependent on whether single or multiple symptoms are assessed; the study’s design (eg, cross-sectional, longitudinal); the research question(s); and/or available statistical expertise. Descriptive statistics (eg, symptom occurrence rates, symptom severity scores) can be used to provide basic information on symptom data. In addition, various parametric and nonparametric tests can be used to describe differences in demographic and clinical characteristics; differences in modifiable and nonmodifiable risk factors; and differences in outcomes between or among individuals with different symptom experiences (eg, sample is divided into individuals with low, moderate, and high pain severity scores using clinically meaningful cutpoints).

While an exhaustive review of the more complex methods for analyzing symptom data is beyond the scope of this paper, some of the newer analytic methods that allow for evaluations of interindividual variability in persons’ symptom experiences and/or interactions within and among symptoms are described below. This information is provided to assist investigators to navigate the conundrum of selecting the most appropriate analytic methods to determine interindividual variability in persons’ symptom experiences. While the choice of analytic methods depends on the research question, study design, and sample size, symptom scientists will need education about these newer and evolving methods. In addition, they will need to develop collaborations with biostatisticians who have expertise in these methods to be able to refine the symptom phenotype(s).

#### Multilevel modeling—

Multilevel modeling also known as hierarchical linear modeling or linear mixed modeling, is a statistical method that analyzes data that varies at multiple levels.^39,40^ In the context of precision health symptom science research, this analytic technique allows for an evaluation of modifiable and nonmodifiable risk factors associated with interindividual variability in the trajectories of a single symptom (for examples see Refs. [41–45]). The first step in this type of analysis is to determine how the symptom changes over time (unconditional model) by testing for linear, quadratic, and cubic trends. Once the shape of the trajectory is determined, one can go through a deliberate process of evaluation of risk factors associated with changes in the intercept (ie, factors associated with the level of the symptom at enrollment) and/or various components of the slope (ie, factors associated with changes in the symptom over time).

For example, if the research question is related to interindividual variability in depression scores over a course of radiation therapy (ie, measured using the Center for Epidemiological Studies Scale (CES-D^46^) that was completed prior to initiation of radiation therapy and monthly for 6 months), the unconditional model provides information on the CES-D score at the start of radiation therapy and how these scores changed over 6 months (eg, increased over time, decreased over time, increased followed by a decrease over time, decreased followed by an increase over time). In addition, one can determine if a prespecified list of risk factors (eg, sex, income, race, ethnicity, body mass index, comorbidity burden, type of cancer, level of exercise, functional status) is associated with higher or lower levels of depression at the initiation of radiation (intercept predictors) and/or with changes in depression scores over time (ie, slope predictors). Of note, and equally important, a variety of biomarkers can be evaluated as predictors of the intercept or slope of specific symptom trajectories.^43,47^

#### Latent variable modeling—

**FIG 2.**
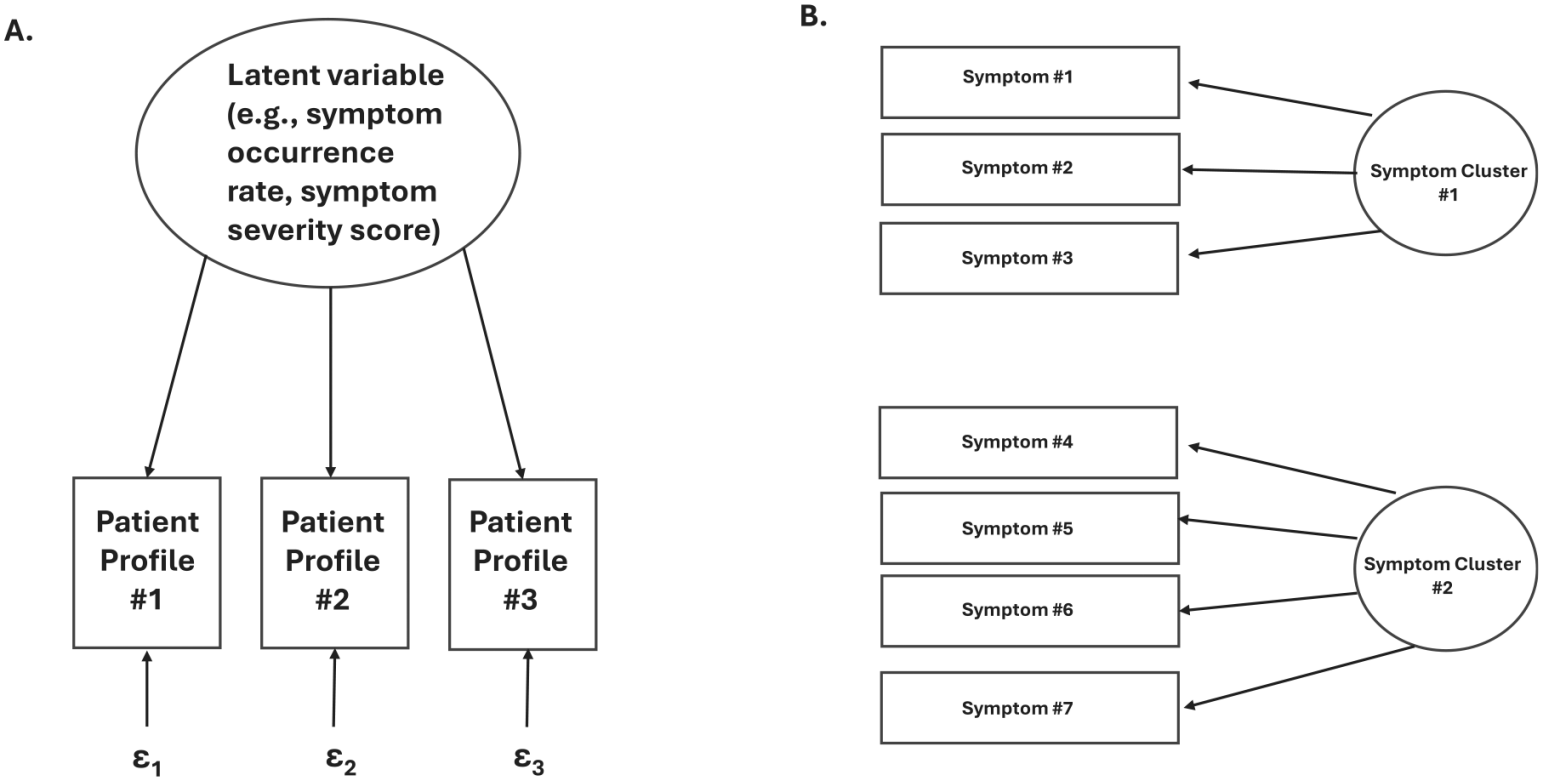
(A) An illustration of a latent variable model of a symptom that identified three distinct patient profiles for that symptom. (B) An illustration of an exploratory factor analysis that identified two symptom clusters using data on seven symptoms.

Latent variable modeling is an analytic technique that links observable variables to unobservable variables. It is a flexible framework that allows for the analysis of different types of data (eg, categorical, continuous, or a combination of the two).^48,49^ Types of latent variable models include latent class analysis (uses categorical variables^50,51^), latent profile analysis (uses continuous scores^52,53^), latent class profile analysis (uses a combination of categorical and continuous scores^54–56^), and latent transition analysis.^56^ In relationship to precision health symptom science research, the observable variable is typically the symptom occurrence rate or the symptom severity or distress score and the unobserved variable is some number of patient subgroups (ie, latent classes) with distinct symptom profiles (Fig. 2A).

This analytic technique is extremely useful to identify individuals within a sample who have very distinct symptom profiles (eg, low, moderate, and high levels of fatigue). Latent variable modeling can be used with cross sectional data (eg, symptoms in a prespecified symptom cluster^57,58^); longitudinal data of single symptoms (eg, pain,^59^ fatigue,^60,61^ sleep disturbance^62^); and longitudinal data with two symptoms (ie, joint latent profile analysis^54,63^ or joint latent class profile analysis). A major advantage of this approach over cluster analysis is that definitive stopping rules also called fit indices (eg, Bayesian Information Criterion, Akaike Information Criterion, Vuong-Lo-Mendell-Rubin test, entropy) are used to determine the optimal number of classes.^64^ Once the final number of classes is determined, modifiable and nonmodifiable risk factors associated with each of the distinct symptom profiles can be evaluated using parametric and nonparametric tests (eg, differences in age, income, and/or functional status among individuals in the low, moderate, and severe pain classes). Equally important, analyses can be done to evaluate for associations between class membership and a variety of biomarkers (eg, candidate genes,^65,66^ changes in gene expression, and pathway perturbations^51,67^).

#### Exploratory factor analysis (EFA)—

EFA is a statistical technique that identifies underlying relationships between variables. It is a data reduction technique that identifies the smallest number of factors that explain the correlations between a set of variables (Fig. 2B).^68^ As noted by Skerman et al,^69,70^ EFA is the preferred method to identify symptom clusters. Initially, defined by Dodd et al,^71^ as “three or more symptoms that are related to each other and may share a common mechanism” (p. 465), over the past 25 years, this important conceptual approach in precision health symptom science research has gained substantial momentum.

The concept of a symptom cluster has clinical validity because across chronic medical conditions, individuals rarely experience only one symptom. A report by an expert panel noted that the concept of a symptom cluster is evolving.^72^ They suggested that, from a conceptual perspective, the defining characteristics of a symptom cluster should include the individual’s symptom experience; temporal characteristics of the symptoms within a cluster; and phenotypic and molecular mechanisms associated with the symptoms within the cluster.^72^ While numerous studies have provided information on symptom clusters in patients with cancer,^11,73–76^ evidence suggests that symptom clusters occur in patients with human immunodeficiency virus,^14,77^ HF,^78,79^ chronic kidney disease,^25,80,81^ and COPD.^82^

From a conceptual perspective, symptom clusters are developed using a “de novo” approach. This approach uses occurrence, severity, or distress data obtained from the administration of a multi-item symptom measure (eg, Memorial Symptom Assessment Scale,^31^ MD Anderson Symptom Inventory^32^). EFA analysis is performed to determine the optimal factor solution (ie, total number of symptom clusters for a given sample). Once the final number of clusters is determined, the clusters are named based on the symptoms within the cluster with the highest factor loadings.

Investigators need to be mindful that the number of symptom clusters that can be identified is dependent not only on sample size, but on the number of symptoms included on the symptom measure used in the study. In addition, evidence suggests that the number and types of symptom clusters identified varies with the dimension of the symptom experience used in the EFA.^75,83–85^ Equally important, the definitions of stability and consistency of symptom clusters, across dimensions of the symptom experience, and across time, have evolved over time. As noted by Harris et al,^11,76^ the original definition proposed by Kirkova and Walsh^86^ suggested that “to be in agreement” or stable, at least 75% of the symptoms within a cluster should be present, including the prominent and most important symptom (ie, symptom with the largest factor loading).

Harris et al noted that while the term “stability” was based on Kirkova and Walsh’s criteria,^86^ the use of the term stability was not consistent across symptom cluster studies.^11,76^ Therefore, Harris et al proposed new definitions and criteria to evaluate the stability and consistency of symptom clusters. The term stability should be used to described whether or not the same clusters are identified across dimensions, time, and/or studies. In contrast, the term consistency should be used to describe whether specific symptoms within a cluster remain the same across symptom dimensions and/or across time.^11,76^ These new definitions of critical concepts in symptom cluster research will assist with comparisons across studies that use the same measures and analytic methods to identify symptom clusters.

#### Network analysis—

**FIG 3.**
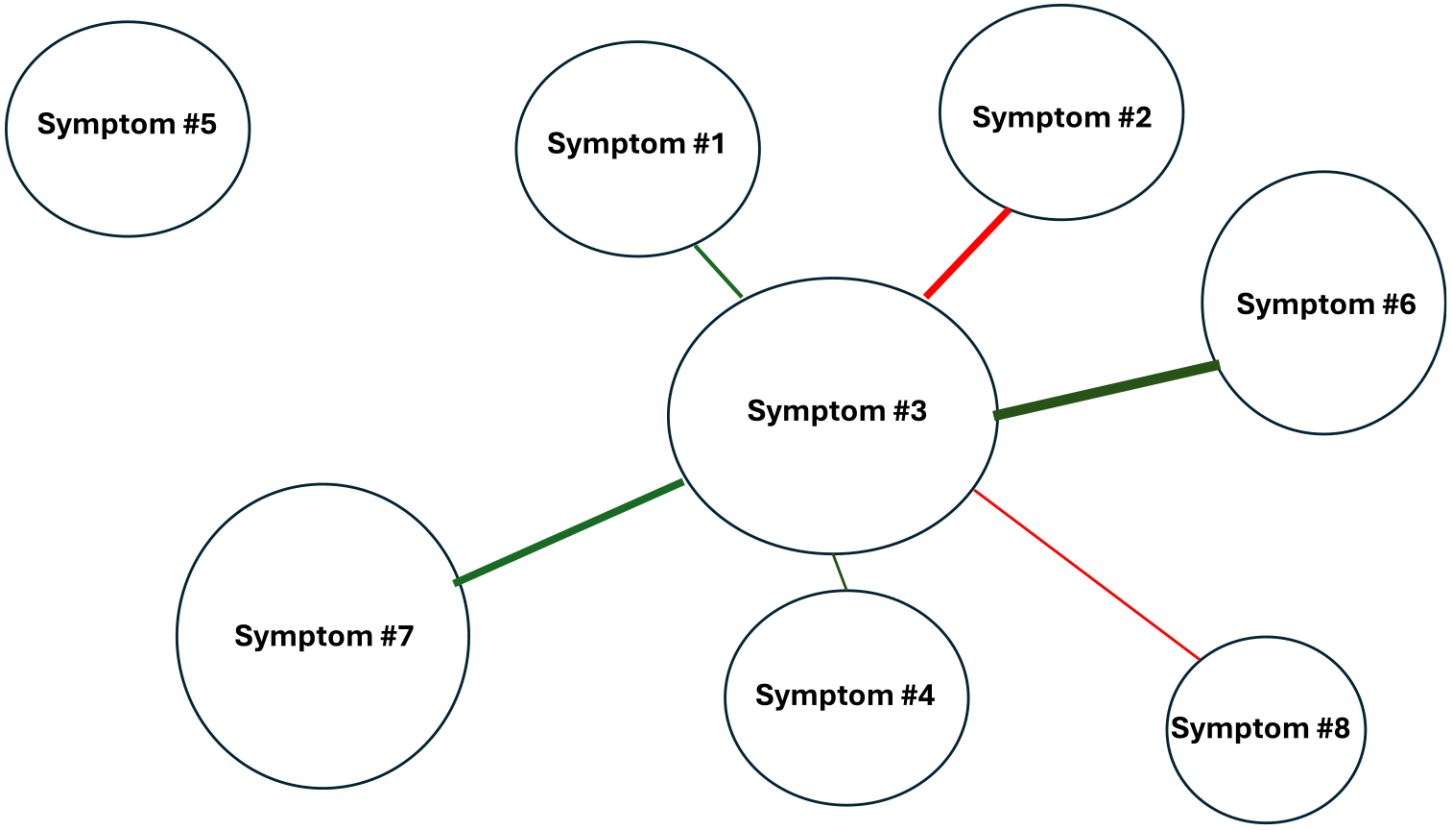
An illustration of a network analysis of eight symptoms. Each oval is a node within the network. The size of the node is proportional to the occurrence rate, severity rating, or distress rating for each symptom. The nodes are connected with interaction links called edges. In terms of the edges, green lines indicate positive connections between symptoms. Red lines indicate negative connections between symptoms. Thicker lines indicate stronger connections. In this example, symptom #5 is not connected within the network.

Network analysis is a graph theory-based approach that allows for analysis of relationships. In terms of precision health symptom science research, network analysis allows for the visualization and quantitative interpretation of the relationships among various symptoms and symptom clusters.^87^ In general terms, networks are defined as a collection of interconnected components (in this paper, symptoms). These components are called nodes, and their interaction links are called edges.^88^ In precision symptom science research, the size of the node is proportional to the occurrence rate, severity rating, or distress rating for each symptom within the network. In terms of edges, green lines indicate positive interconnections between symptoms. Red lines indicate negative interconnections. Thicker lines indicated stronger connections (Fig. 3).^87^

While symptom clusters can be identified using EFA, the inter-relationships among symptoms within a cluster; the inter-relationships among symptoms across clusters; and the inter-relationships among the symptom clusters themselves cannot be determined. In addition, sentinel or core symptoms within clusters cannot be identified. Network analysis of symptoms provides new insights into the strength of relationships among symptoms within a network; as well as which symptoms are not connected in a network. In addition, if symptom clusters within a network are identified, the relationships among the various symptom clusters can be visualized.

While a variety of methods exist to perform network analyses, one of the advantages of this analytic approach is the availability of centrality indices. These indices provide insights into the structural importance of each node within a network.^89,90^ Betweenness, closeness, and strength are the three most commonly estimated centrality indices. Betweenness measures the number of times a node lies on the shortest path between two other nodes. This index suggests which nodes act as bridges between other nodes in the network. Closeness is a summary of the average distance of one specific node to all the other nodes in the network. Closeness allows for the identification of nodes (ie, symptoms) that are in a position to have a substantial influence on other node(s) (ie, other symptom(s)) in the network. Strength provides information on which node has the strongest overall connections within a network. It is calculated by summing the absolute edge weights that are connected to a specific node. Strength provides information on the most connected node (ie, symptom) inside a network. Nodes with high centrality indices are considered core nodes in the network (ie, sentinel or core symptoms).^87^

The use of network analysis is gaining momentum in precision health symptom science research. For example, this analytic method was used to identify the relationships among symptoms in patients with cancer,^87,91^ HF,^92,93^ and COPD.^94^ Other applications for network analysis, that can be done within the context of precision health symptom science research, include: the identification of genes associated with various chronic conditions using molecular interaction networks^95,96^; drug discovery and repurposing of existing medications^97–99^; patient stratification using genomic data, demographic and clinical information, and symptom burden; identification of biomarkers and mechanisms associated with a higher symptom burden using individual symptom scores and/or symptom cluster scores; and the development of personalized symptom management interventions using demographic and clinical characteristics, symptom data, and molecular data.

#### Artificial intelligence and machine learning—

Artificial intelligence is a broad concept that includes machine learning as a subset. Artificial intelligence can be defined as the ability of a machine to mimic human intelligence (ie, think, learn, and act like a human). Machine learning is the development and use of computer systems that are able to learn and adapt without following explicit instructions, by using algorithms and statistical models to analyze and draw inferences from patterns in data.^100–107^ In most machine learning paradigms, the data are divided into two groups, a “training” or “discovery” data set and a “test” or “validation” data set. Therefore, sample sizes warrant careful consideration when these methods are employed.

While an exhaustive discussion of artificial intelligence and machine learning is beyond the scope of this paper, suffice it to say—their use in precision health symptom science research will evolve and grow. Scientists, with expert knowledge of the symptom and/or medical condition, will need to evaluate the validity and reproducibility of various artificial intelligence and machine learning models in the context of symptom assessment and management. Of particular importance will be to determine the accuracy of various machine learning methods to predict symptom occurrence and/or severity, as well as symptom burden.

### Collection of “Meaningful” Data

The word meaningful or “full of meaning” comes from the word mean that has its roots in the Old English word mænan that translates to “to signify, tell, or complain.”^108^ Precision health symptom scientists have an abundance of sources to use to collect meaningful data. The source of the symptom data will be influenced by the study’s purpose and design; the availability of specific data sources; the cost-benefit ratio for obtaining the data; and the expertise of the research team who is responsible for the management and analysis of different types of data.

#### Paper versus electronic questionnaires—

To date, most precision health symptom science research obtained self-report data from individuals. While traditional approaches used questionnaire booklets; data collection using electronic tablets, computer-based questionnaires, and/or smartphones are becoming more common. As noted in a recent review,^109^ some of the advantages of using web-based applications include: the possibility of recruiting a geographically diverse sample in a shorter period of time; decreased financial costs; and greater flexibility and decreased participant burden. However, limitations with the use of this approach do exist, including: a potential lack of inclusiveness in study participants; lack of control over who completes the questionnaire(s); a chance of decreased comprehension of the content or intent of the study questionnaires; and a potentially lower level of participant engagement.

In terms of participant preferences for paper versus web-based formats, findings from two studies are worth noting.^110,111^ In a study of Australian women who were given the choice to complete study instruments using a paper questionnaire or a web-based application (computer, tablet, or phone),^110^ the authors reported that the online option was more likely to be chosen by better educated and healthier women and most of these women used a computer to complete the measures. The authors concluded that if mixed-mode options are feasible, they are more likely to produce more representative results than only an online option. In another study that compared paper to electronic questionnaires,^111^ patients who were younger and who had a higher level of education chose the electronic format. In addition, the authors noted that when gathering data in the hospital, the tablet was convenient and was associated with very little missing data. In contrast, when filling out questionnaires at home, patients preferred a paper questionnaire. These considerations are important because they can influence participation rates and data integrity.

#### Use of the electronic health record (EHR)—

While medical records have existed since the time of Hippocrates,^112^ the ability to access deidentified data from the EHR will have a significant impact on precision health symptom science research. The EHR represents a source of “real world data” on a potentially large number of individuals. Advocates note that the use of this type of data can lead to improved patient outcomes and the development of clinical decision support systems.^113^ However, to be useful in precision health symptom science research, the assessment of symptoms must be done using a standardized approach.

As noted by Koleck et al,^114^ symptom information often requires manual extraction from clinicians’ notes, which is laborious, time-consuming, and prone to error. However, using a natural language processing application, her team was able to extract symptom information from EHR notes in an accurate and scalable manner. In a more recent study that used natural language processing of clinical notes to identify symptoms in oncology patients,^115^ predictive models for the development of 12 common symptoms were identified using structured and unstructured EHR data. While the factors that predicted symptom occurrence varied by symptom, they included a variety of patient demographic and clinical characteristics, as well as treatments.

The best methods for working with the EHR, as a source of “real world” data, warrant ongoing evaluation. Precision health symptom scientists will need to develop collaborations with clinicians; experts in biomedical informatics and information technology; biostatisticians with expertise in data science, artificial intelligence, and machine learning; ethicists; and individuals with the symptom(s) and medical condition(s) to optimize the use of this source of data. In addition, the incorporation of symptom measures into the EHR and the routine collection of these data in inpatient and outpatient settings will facilitate precision health symptom science research.

#### Use of public databases—

A variety of large databases are available free of charge to conduct precision health symptom science research. Two examples include the All of Us Research Program^116^ and the UK Biobank.^117^ In brief, started in 2018, the All of Us Research Program plans to enroll a diverse group of at least one million persons in the United States in order to accelerate biomedical research and improve health. This database includes health questionnaires, EHR data, physical measurements, and the collection and analysis of biospecimens. The overall goal of this data repository is to allow researchers to account for interindividual differences in lifestyle and socioeconomic factors, as well as environmental and biological characteristics, to advance precision diagnoses, prevention efforts, and treatment strategies.

In terms of the symptom data within the All of Us database, in the overall health questionnaire, participants are asked to rate their pain on average, fatigue level, and bother from emotional problems (ie, feeling anxious, depressed, or irritable). Under the conditions section, several medical conditions associated with symptoms are evaluated (eg, pain, musculoskeletal pain, joint pain, neuropathy, anxiety, sleep disorder, anxiety disorder, cough, difficulty breathing, fatigue, dyspnea, dizziness). While precision health symptom scientists need to register to gain access to this resource and may require education related to using the online database and analysis tools, this public database is a valuable resource.^116^ Additional information on the All of Us Program can be found at https://allofus.nih.gov.

The UK Biobank is a large-scale biomedical database and resource for scientists that contains deidentified genetic, lifestyle, and health information and biological samples from half a million participants from the United Kingdom (see https://www.ukbiobank.ac.uk). Online questionnaires provide data on pain, cognitive function, mental health, visualization and memory, and sleep. Researchers need to register and apply for access to the data based on a summary of their research project.^117^ These two public databases, as well as numerous others available from the Centers for Disease Control and Prevention, National Center for Health Statistics, Medicare and Medicaid, and other federal and state agencies in the United States are valuable resources for precision health symptom science research (for a list of some of these potentially useful datasets see—https://hsls.libguides.com/health-data-sources/data-sets).

#### Qualitative data—

Given the state of the science in precision health symptom science research, a need exists to conduct qualitative and mixed methods studies to obtain in-depth knowledge of individuals’ symptom experiences. Qualitative research is a type of research that explores and provides deeper insights into real-world problems. In brief, scientists focus on individuals’ experiences, perceptions, and behaviors. Using open-ended questions, findings from qualitative research have the ability to explain processes and patterns of human behavior that are difficult to quantify.^118^

Equally important, a mixed methods approach to precision health symptom science research may be an optimal method for many studies. As the name implies, it combines elements of quantitative and qualitative research to answer the study’s specific aims. This approach helps scientists to gain a more complete picture of the topic under investigation.^119,120^ For example, in precision health symptom science research, the use of qualitative or mixed methods research may increase one’s understanding of specific symptoms,^121,122^ the concept of a symptom cluster,^123^ and/or the efficacy of a symptom management intervention.^124–127^

## Potential Challenges/Opportunities in Precision Health Symptom Science Research

While significant progress has occurred in precision health symptom science research over the past two decades, it remains a relatively young field of scientific inquiry. Therefore, several challenges/opportunities deserve consideration. While this list is not exhaustive, it provides a roadmap of important next steps.

One potential challenge in doing this type of research is to become discouraged with the list of conundrums summarized in this paper. However, all of them are opportunities to move precision health symptom science forward. All of the conundrums can be used to guide a scientist’s program of research. Numerous opportunities exist to answer fundamental questions related to interindividual differences in persons’ symptom experiences that form the basis for precision health symptom science research (ie, detailed characterization of the symptom phenotype and development of individualized interventions to prevent and/or treat symptoms).

A central question that warrants careful consideration is how much and what types of data to collect in a single study. While this paper focused on the characterization and analysis of the symptom phenotype, other papers in this issue will provide insights into other types of data. The detailed characterization of the symptom phenotype is the most important component of a precision health science symptom study. That said, scientists will need to make difficult choices about the collection of other types of data. If the research question(s) is focused on the elucidation of underlying mechanisms, careful consideration needs to be given to the type(s) of biomarker data that will be collected (eg, genome, epigenome, transcriptome, proteome, metabolome, inflammasome) because each of these biomarkers provides information on different biological functions.

Given the growing evidence on associations between a wide variety of symptoms and alterations in the microbiome,^128–132^ this biomarker may be an important one to evaluate. However, scientists need to determine the best site for the collection of the specimen (eg, nose, mouth, lungs, stomach, colon, sexual organs, skin) based on the symptom of interest, because at each of these sites, the microbiome has different functional consequences.^133^ Equally important, if the research question is focused on a determination of the correlation between the symptom phenotype and objective measures of the symptom [if available], consideration needs to be given to the source and expense associated with this type of data collection (eg, magnetic resonance imaging, activity monitoring, wearable devices, home sensors, ultrasound, echocardiography). In addition, the collection of specimens needs to be standardized, and proper storage of specimens ensured, particularly with multisite studies.

One final potential challenge/opportunity in precision health symptom science research is the need to consider significant covariates that may contribute to the occurrence and severity of individual symptoms and overall symptom burden. While not an exhaustive list and undoubtedly subject to growth, specific covariates that warrant evaluation include a variety of SDOH (eg, economic stability, access to health care, neighborhood and built environment, nutritional status)^134^; epigenetic age acceleration^135,136^; and stress, particularly adverse childhood experiences.^137,138^ Given that the literature on the influence and/or impact of these covariates on individuals’ symptom experiences and symptom burden is growing at an exponential rate, this potential challenge/opportunity needs to be addressed in future research studies.

Given the finite amount of resources and a concern about participant burden, precision health symptom scientists need a plan to maximize data collection, particularly with individuals in the clinical setting. If resources do not allow for the processing and analysis of biological specimens, these samples can be collected and stored for future use. In terms of respondent burden, some questionnaires can be administered at different times during the course of the study to optimize data collection; obtain a more complete picture of potential covariates; and facilitate the collection of complete data. In addition, if an intervention is part of a precision health symptom science study, consideration should be given to recording the interactions between the interventionist and the participant. These audio recordings can be analyzed at a future date using qualitative or mixed-method approaches. They may provide valuable information on the most effective components of an intervention; components that require modifications to be more effective; components that can be eliminated; and/or components that need to be added in future studies.

In order to optimize the analysis of data, precision health symptom scientists will need to develop transdisciplinary collaborations and research teams with members who have different and diverse areas of expertise. Precision health symptom science research is the ultimate example of the need for team science. The inclusion of scientists with different areas of expertise provides the space for new ideas and varied points of view and enhances the quality of the study. Equally important, precision health symptom scientists will need to increase their knowledge and skills in a number of areas (eg, biomarkers, big data analytics, artificial intelligence). While in-depth expertise is needed on the research team, precision health symptom scientists will need a “working knowledge” of these new areas to be able to engage in meaningful dialogue with team members.

## Critical Directions and Recommendations for Future Research

The future of precision health symptom science research is both exciting and challenging. Each scientist needs to develop an individual plan and program of research. This plan needs to include the symptom(s) that will be investigated; the measures that will be used to determine the symptom phenotype; the covariates that warrant evaluation; the types of biomarkers that will be collected; and/or the specific objective measures that will be assessed. While not every study will have the resources to collect every single piece of data, the science will grow exponentially if an individual investigator uses the same core measures across his/her studies. This approach will allow for replication and/or verification of study findings.

Table 3 provides some recommendations for future directions for precision health symptom science research. While not exhaustive, this list contains suggestions for research studies that will move the field of precision health symptom science forward. Given that the specific symptoms and the amount of symptom burden are relatively ubiquitous across medical conditions (Tables 1 and 2), the next generation of precision health symptom science studies should have as a goal comparisons across a variety of acute and chronic medical conditions. The ultimate goal of all of the descriptive and correlational science in this field is to develop, test, and implement personalized interventions to prevent and/or treat symptoms across one or more acute or chronic medical conditions.

## Limitations and Overall Conclusions

While this paper provides an overview of the conundrums and challenges in precision health symptom science research, several limitations warrant consideration. This manuscript represents a narrative review of the literature. The conclusions and recommendations presented in this paper are based on expert synthesis and interpretation of the extant literature. In fact, each section of this paper is worthy of a scoping review and/or a systematic review and meta-analysis. Some of the recommendations contained in this review (eg, use of various analytic methods, use of biomarkers) will require that symptom scientists gain new knowledge and skills and develop collaborations with experts in these fields.

Despite these limitations, this paper makes some unique contributions to the literature, including: a synthesis of cutting-edge analytic techniques, the proposed integration of various symptom science research methods across common medical conditions; and clearly defined research recommendations that can drive precision health symptom science research for the next few decades. The authors hope that this paper will serve as a reference tool for graduate students and researchers. In addition, the contents can be used to guide the development of curriculums in precision health symptom science research.

## Figures and Tables

**TABLE 1:** Common Symptoms Across Chronic Medical Conditions

Symptom	COPD^13^	CKD^15^	HF^12^	T2DM^16^	HIV^14^	Cancer^11^
Fatigue	□	□	□	□	□	□
Pain	□	□	□	□	□	□
Sleep disturbance	□	□	□	□	□	□
Cognitive changes	□	□	□	□	□	□
Depression	□	□	□	□	□	□

CKD, chronic kidney disease; COPD, chronic obstructive pulmonary disease; HF, congestive heart failure; HIV, human immunodeficiency virus; T2DM, type 2 diabetes mellitus.

**TABLE 2:** Number of Symptoms Across Chronic Medical Conditions

Chronic condition	Number of symptoms
Chronic obstructive pulmonary disease^13^	1.9 (±2.1) to 17.6 (±5.4)
Heart failure^27^	15.1 (±4.9)
Chronic kidney disease^26^	6.3 (±2.5) to 17.9 (±4.4)
Type 2 diabetes mellitus^16^	2.0 (±1.9) to 4.5 (±2.3)
Human immunodeficiency virus^14^	9.7 (±5.4)
Cancer^11^	13.9 (±7.2)

**TABLE 3:** Recommendations for Future Research

Measurement of symptoms
□ Determine the best measures to evaluate the most common single symptoms within and across acute and chronic medical conditions □ Determine the core symptoms that need to be included in an instrument to evaluate multiple symptoms within and across acute and chronic medical conditions □ Evaluate for differences within and among common acute and chronic medical conditions in various dimensions of the symptom experience (eg, occurrence, frequency, severity, distress) □ Develop methods to determine symptom burden in patients with acute and chronic medical conditions
Interindividual differences in patients’ symptom experiences
□ Compare and contrast the optimal methods to characterize single and multiple symptoms (ie, breadth or depth in determining the symptom phenotype) within and across acute and chronic medical conditions □ Determine occurrence rates for the most common single and multiple symptoms within and across acute and chronic medical conditions □ Compare and contrast the severity and distress ratings for the most common single and multiple symptoms within and across acute and chronic medical conditions
Concept of “Time” in the evaluation of symptoms
□ Determine the optimal timing for the administration of symptom measures within and across acute and chronic medical conditions to maximize the detection of changes □ Determine the optimal timing for the administration of symptom measures during an intervention study to be able to determine when treatment efficacy occurs and whether efficacy is maintained
Data analytic approaches
□ Determine, within and across acute and chronic medical conditions, the optimal modifiable and nonmodifiable risk factors associated with initial levels and trajectories of common single symptoms □ Determine, within and across acute and chronic medical conditions, the number and types of symptom(s) profiles that are associated with various aspects of care and/or across the continuum of the condition’s clinical course □ Compare and contrast the number and types of symptom clusters within and across common acute and chronic medical conditions □ Determine the optimal number of common symptoms to include in a questionnaire to evaluate symptoms clusters within and across acute and chronic medical conditions □ Compare and contrast symptom networks within and across acute and chronic medical conditions □ Determine the optimal number of common symptoms to include in a questionnaire to evaluate symptoms networks within and across acute and chronic medical conditions □ Compare and contrast the core or sentinel symptoms within a network within and across acute and chronic medical conditions □ Determine the best machine learning methods to use to predict single symptom trajectories and/or symptom burden within and across acute and chronic medical conditions
Collection of “Meaningful” data
□ Determine participant characteristics that influence an individual’s preferences for the mode of administration for symptom questionnaires □ Compare and contrast, within and across acute and chronic medical conditions, the number and types of symptom data that can be extracted from the electronic health record □ Determine the best machine learning methods to use to predict single symptom trajectories and/or symptom burden within and across acute and chronic medical conditions using data extracted from the electronic health record □ Identify the types of precision health symptom science research studies that can be done using one or more public databases □ Compare and contrast, within and across acute and chronic medical conditions, using qualitative methods, individual’s perceptions of their symptom experiences □ Determine the influence of various social determinants of health and evolving covariates (eg, epigenetic age acceleration, stress, adverse childhood experiences) on the occurrence and severity of single and multiple symptoms within and across acute and chronic medical conditions) □ Determine the most accurate types of biomarkers to use to predict the occurrence and trajectories of single and multiple symptoms within and across acute and chronic medical conditions □ Compare and contrast, within and across acute and chronic medical conditions, the common underlying mechanism(s) for single and multiple symptoms □ Determine the common and distinct mechanisms associated with subjective and objective measures of single symptoms within and across acute and chronic medical conditions

## Layperson Summary

### What we investigated and why

Across common medical conditions, unrelieved symptoms have a negative impact on all aspects of a person’s life. Therefore, a narrative summary of the state of the “art” of precision health symptom science research was needed.

### How we did our research

A review of the literature was done, and experts synthesized and interpreted the findings.

### What we have found

Precision symptom science research is a young field of scientific inquiry. Scientists need information on how to conduct studies of symptoms in individuals with one or more common medical conditions (for example, cancer and/or heart failure).

### What it means

Numerous opportunities exist to conduct high-quality research that will improve symptom management through the development of personalized interventions.
